# Danggui Sini decoction for treating diabetic peripheral neuropathy: A protocol of systematic review and meta-analysis of randomized controlled trials

**DOI:** 10.1097/MD.0000000000020482

**Published:** 2020-05-22

**Authors:** Xiyu Zhang, Heting Wang, Yuan Zhang, Ya Liu, Zhenxing Wang, Quanyu Du, Chunguang Xie

**Affiliations:** a Hospital of Chengdu University of Traditional Chinese Medicine; b Sichuan Provincial People's Hospital, Chengdu, Sichuan Province, China.

**Keywords:** Danggui Sini decoction, diabetic peripheral neuropathy, protocol, systematic review

## Abstract

**Background:**

Diabetic peripheral neuropathy (DPN) is one of the most common chronic complications of diabetic patients, which seriously affects the quality of life of patients. At present, mainstream drugs have problems such as poor efficacy and side effects. Traditional Chinese medicine (TCM) has extensive clinical experience in the prevention and treatment of diabetes and chronic complications, and it also shows clear advantages in the treatment of DPN. Clinical studies have confirmed that Danggui Sini decoction (DSD), a TCM decoction, can improve the clinical symptoms and signs of DPN patients. Therefore, we will conduct a systematic review to clarify the effectiveness and safety of DSD for DPN.

**Methods:**

We will search every database from the built-in to October 2020. Chinese literature comes from CNKI, Wanfang, VIP, and CBM databases. English literature mainly searches Cochrane Library, PubMed, Web of Science, and EMBASE. At the same time, we will also search for clinical registration tests and gray literatures. This study only screened clinical randomized controlled trials (RCT) for DSD for DPN. The two researchers independently conducted literature selection, data extraction and quality assessment. Dichotomous data is represented by relative risk (RR), continuous data is represented by mean difference (MD) or standard mean deviation (SMD), and the final data is fixed effect model (FEM) or random effect model (REM), depending on whether it exists Heterogeneity. The main result is clinical efficacy and nerve conduction velocity. Fasting blood glucose, 2 hours postprandial blood glucose, blood lipid, hemorheology, and adverse events are secondary results. Finally, a meta-analysis was conducted through Review Manager software version 5.3.

**Results:**

This study will conduct a comprehensive analysis based on the currently released DSD data for the treatment of DPN and provide high-quality evidence of clinical efficacy and safety.

**Conclusion:**

This systematic review aims to provide new options for DSD treatment of DPN in terms of its efficacy and safety.

**Ethics and dissemination:**

The review is based solely on a secondary study of published literatures and does not require ethics committee approval. Its conclusion will be disseminated in conference papers, magazines, or peer-reviewed journals.

**INPLASY registration number:**

INPLASY202040157.

## 1 Introduction

Diabetic peripheral neuropathy (DPN) is one of the most common chronic complications of diabetes and the most common form of neuropathy worldwide,^[1]^ with sensory and autonomic symptoms as the main clinical manifestations. DPN is defined as, “the presence of symptoms and/or signs of peripheral nerve dysfunction in people with diabetes after the exclusion of other causes”.^[2]^ The prevalence of peripheral neuropathy in diabetic adults is estimated to be between 6% and 51% according to different factors such as age and time of diabetes.^[3]^ DPN eventually affects nearly 50% of adults with diabetes during their lifetime and is associated with substantial morbidity including pain, foot ulcers, and lower limb amputation. The quality of life of patients with DPN has been significantly affected physiologically and psychologically,^[4]^ and 43% of the patients reportedly have anxiety, depression, and sleep disorders.^[5]^

Currently, the treatment of DPN mainly includes blood glucose control, foot care and pain management. However, the evidence that blood glucose control can effectively reduce the symptoms of patients with peripheral neuropathy is still insufficient.^[6]^ Studies have shown that antioxidants, neurotrophic drugs, aldose reductase inhibitors, etc can be used to treat DPN, but there are disadvantages of toxic side effects and poor tolerance.^[7]^ Therefore, for DPN, it is necessary to seek a more effective treatment.

In recent years, the advantages of Traditional Chinese medicine (TCM) in preventing and treating such chronic diseases have been gradually recognized worldwide. Danggui Sini decoction (DSD) is a classical prescription described in Shanghan Zabing Lun, written by Zhang Zhongjing during the Han dynasty. DSD is composed of Angelica sinensis (Danggui), Cassia twig (Guizhi), Peony (Shaoyao), Asarum (Xixin), Mushroom (Tongcao), Licorice (Gancao), Jujube (Dazao), all of which are reported to nourish Qi, and to remove pathogenic factors such as wind cold and damp, warming and smoothing meridians, and invigorating the blood to promote coronary circulation. In general, HGWD is used to treat blood impediments, characterized by feelings of pain and numbness, muscle discomfort, muscular atrophy, consistent with the features of DPN. Studies have shown that DSD has the effect of improving nerve pain, and its mechanism is related to the inhibition of neuroinflammation in the dorsal horn of the spinal cord, the influence of platelet aggregation, and the expression of tissue factors and fibrinase.^[8,9]^

## 2 Methods

### 2.1 Protocol registration

The systematic review protocol has been registered on the INPLASY website as INPLASY202040157 (https://inplasy.com/inplasy-2020-4-0157/). Strictly follow the guidelines of Cochrane Handbook for Systematic Reviews of Interventions and the Preferred Reporting Items to conduct this Systematic Reviews and Meta-analysis Protocol (PRISM-P),^[10]^ and record important program revisions in the complete evaluation.

### 2.2 Inclusion criteria

#### 2.2.1 Study design

The study selected only clinical randomized controlled trials of DSD against DPN published in Chinese and English. Animal mechanism studies, reviews, case reports, and nonrandomized clinical trials will be excluded.

#### 2.2.2 Participants

DPN patients, regardless of race, gender, and age, must meet the diagnostic criteria of the “Chinese Type 2 Diabetes Prevention and Treatment Guidelines” issued by the Diabetes Branch of the Chinese Medical Association in 2017.^[11]^ Neuropathy caused by other reasons, patients with severe cardiovascular and cerebrovascular diseases, severe liver and kidney dysfunction, mental illness, related drug allergies, pregnancy and lactation patients will not be included.

#### 2.2.3 Interventions

Both groups were cured with conventional diabetes treatments recommended by the ADA guidelines, including diet, exercise, and hypoglycemic and lipid-lowering therapies.^[12]^ The experiment group used DSD or modified DSD, while the control group applied for placebo, or no treatment. In addition, the two groups did not take any drugs that interfered with the outcome indicators. The follow-up time was ≥4 weeks.

#### 2.2.4 Outcomes

The primary outcomes include the improvement in clinical efficacy and nerve conduction velocity. The clinical efficacy refers to the guiding principles for clinical research of new Chinese medicines^[13]^ and is determined according to the degree of improvement of the symptoms of the patient before and after treatment: markedly effective: the clinical symptoms and signs of TCM improved significantly more than 70%; effective: the clinical symptoms and signs of TCM reduced by 30% to 70%; Ineffective: the clinical symptoms and signs of TCM improved less than 30% or no improvement, or even worse. The nerve conduction velocity includes the sensory nerve conduction velocity and the motor nerve conduction velocity, which are evaluated by electromyography.

Secondary outcomes are mainly composed of fasting blood glucose, 2 hours postprandial blood glucose, blood lipid, hemorheology, and adverse events.

### 2.3 Search methods

#### 2.3.1 Electronic searches

We will retrieve each database from the built-in database until October 2020. Chinese literature comes from CNKI, Wanfang, VIP, and CBM databases. English literature mainly searches Cochrane Library, PubMed, Web of Science, and EMBASE. We adopt the combination of heading terms and free words as search strategy, which decided by all the reviewers. Search terms: Danggui Sini Decoction, Danggui Sini Tang, Danggui Sini, Dang Gui Si Ni, diabetes mellitus, diabetes, diabetic, diabetic neuropathies, diabetic peripheral neuropathy. We will simply present the search process of the Cochrane Library (Table 1). Adjusting different search methods according to different Chinese and English databases.

**Table 1:**
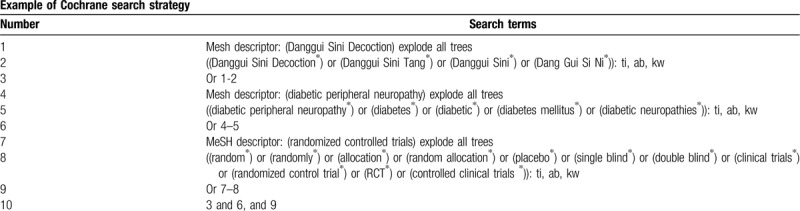
Cochrane Library search strategy.

#### 2.3.2 Searching other resources

At the same time, we will retrieve other resources to complete the deficiencies of the electronic databases, mainly searching for the clinical trial registries and gray literature about DSD for DPN on the corresponding website.

### 2.4 Data collection and analysis

#### 2.4.1 Selection of studies

**Figure 1:**
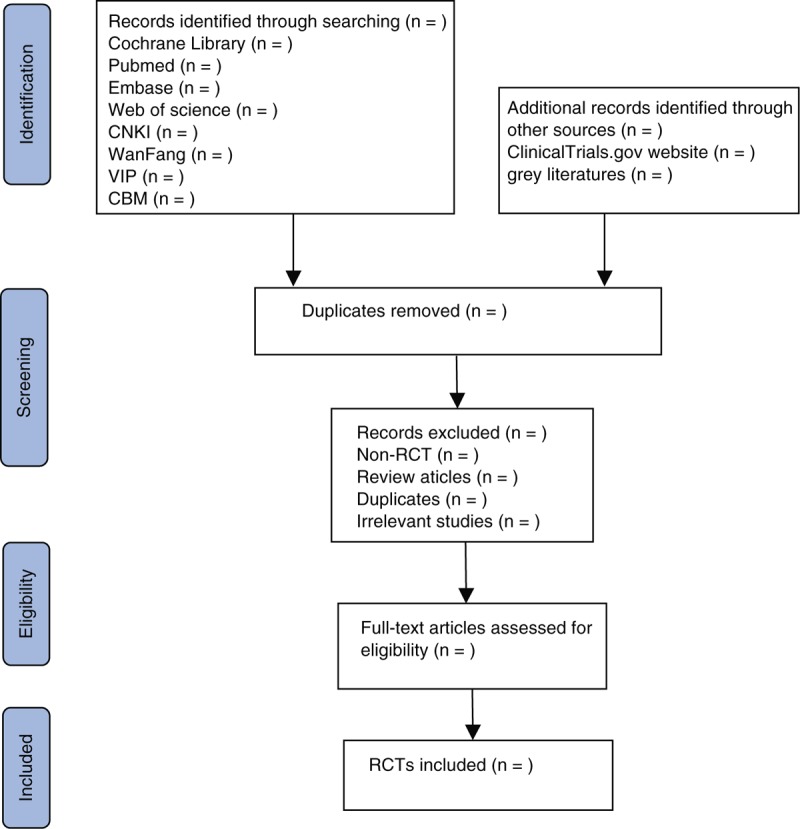
Flowchart of the study selection.

Two reviewers will independently search all documents. The references identified from relevant database searches will be imported into the EndNote X9 software. After that, two independent reviewers preliminarily screened the literature that may meet the established criteria of the study by reading the title and abstract, and then reviewed the full text to confirm their inclusion. If there are any disagreements, the two researchers will discuss and reach an agreement. If no consensus can be reached, a third-party will be consulted to reach an agreement. A flowchart (Fig. 1) will be used to describe the identification and selection process of the study.

#### 2.4.2 Data extraction and management

According to the eligibility criteria, the two reviewers will use the same eligibility assessment form to evaluate the research, and the results of the qualified research will be independently extracted. The main data extracted are as follows: study characteristics (title, author, year, fund source), participant characteristics (sample size, gender, age, duration of disease), intervention details (medicine, dose, frequency, duration), outcomes (clinical cure rate, adverse reactions), etc. The above information was finally cross-checked by two reviewers. When the reported data is insufficient or ambiguous, a researcher will contact the corresponding author via phone or email for more information.

#### 2.4.3 Assessment of risk of bias

The investigator will evaluate all included studies in accordance with the guidelines of the Cochrane Handbook for Systematic Reviews of Interventions. The following items related to the risk of bias, including random sequence generation, allocation concealment, blind participants and personnel, blind assessment of results, incomplete result data, selective result reports, and other biases, will be evaluated by two reviewers. The quality of each trial is classified as “low”, “high”, or “unclear” risk of bias. The discrepancies will get a consistent conclusion by discussing between both reviewers or seeking the third-party consultation.

#### 2.4.4 Measures of treatment effect

Select the evaluation method according to different curative effect indexes. For the dichotomous data, we will choose the relative risk (RR) of the indicator of the effect scale with 95% confidence interval (CI). For continuous data expressed as mean difference (MD) or standard mean difference (SMD), the CI value is 95%, depending on whether the measurement range is consistent.

#### 2.4.5 Dealing with missing data

If the missing relevant data is still not available after contacting the author, we can synthesize the available data in the initial analysis. In addition, sensitivity analysis will be used to assess the potential impact of missing data on the overall results of the study.

#### 2.4.6 Data analysis

We use Review Manager software version 5.3 provided by Cochrane Collaboration to analyze the data. The RR of 95% CI was used to summarize the dichotomous data. Continuous data will be summarized by using the weighted MD of 95% CI. According to research recommendations,^[14]^ we will use a random effect model (REM) for meta-analysis in this paper.

Statistical heterogeneity will be evaluated by Chi-square test and *I*^2^ test. *P*-Value ≥ .1 and *I*^2^ ≤ 50% indicate that the study has no significant statistical heterogeneity. In contrast, *P*-value < .1 or *I*^2^ > 50%, indicating that there is considerable heterogeneity. When there is no statistical heterogeneity, a fixed effect model (FEM) will be used. In contrast, when there is a statistical heterogeneity, a REM will be used. In addition, we will conduct subgroup or sensitivity analysis to find potential causes. If meta-analysis cannot be performed, we will conduct a descriptive analysis.

#### 2.4.7 Subgroup analysis

We will conduct subgroup analysis based on different reasons such as age, gender, different forms of intervention, drug dosage form, dosage, treatment process, etc.

#### 2.4.8 Sensitivity analysis

To evaluate the robustness of the meta-analysis results, we will first delete the low-quality studies and then merge the data to assess the impact of the sample size, study quality, statistical methods, and missing data on the meta-analysis results.

#### 2.4.9 Reporting bias

If there are more than 10 studies in the meta-analysis, the symmetry of the funnel plot will be assessed to examine publication bias, with results being interpreted cautiously.

#### 2.4.10 Grading the quality of evidence

The investigator will use “the Grading of Recommendations Assessment, Development and Evaluation system (GRADE)” to independently assess the quality of evidence for each result.^[15]^ The GRADE system divides the quality of evidence into four levels: high, medium, low, and very low. GRADE profiler 3.2 will be used for analysis.

## 3 Discussion

DPN affects approximately 50% of diabetic patients. Manifestations of pain, numbness, paresthesia, and ulcers in the extremities are the main causes of non-traumatic amputation.^[16]^ At present, mainstream drugs have problems such as poor efficacy and large side effects.^[17,18]^ Therefore, clinicians and patients urgently need to find a more effective treatment.

TCM has been used to treat diabetes and diabetes complications for many years in China. Clinical practice shows that DSD can alleviate the symptoms of DPN and improve clinical efficacy.^[19,20]^

At present, there is no evidence-based medicine to confirm the efficacy of DSD on DPN. Therefore, we try to conduct this meta-analysis to provide high-quality evidence on the clinical efficacy and safety of DSD, and hope to promote the application of TCM and benefit more patients.

## Author contributions

**Conceptualization:** Xiyu Zhang, Chunguang Xie.

**Data curation:** Heting Wang, Yuan Zhang.

**Formal analysis:** Yuan Zhang, Ya Liu.

**Funding acquisition:** Chunguang Xie.

**Methodology:** Zhenxing Wang, Quanyu Du.

**Project administration:** Chunguang Xie, Heting Wang.

**Resources:** Xiyu Zhang, Ya Liu, Chunguang Xie.

**Software:** Xiyu Zhang, Ya Liu, Chunguang Xie.

**Supervision:** Heting Wang, Yuan Zhang, Ya Liu.

**Writing – original draft:** Xiyu Zhang, Heting Wang.

**Writing – review & editing:** Chunguang Xie.
